# The Relationship Between Orthodontic Malocclusion Severity and Periodontal Clinical and Biochemical Parameters in Adolescents: A Cross-Sectional Study

**DOI:** 10.3390/diagnostics16182929

**Published:** 2026-09-10

**Authors:** Nur Yorgancilar, Tuba Kose, Samet Uygun, Oguz Kose, Kerimali Akyildiz, Adnan Yilmaz

**Affiliations:** 1 Department of Periodontology, Faculty of Dentistry, Recep Tayyip Erdoğan University, 53020 Rize, Turkey; oguz.kose@erdogan.edu.tr; 2 Department of Orthodontics, Faculty of Dentistry, Recep Tayyip Erdoğan University, 53020 Rize, Turkey; tuba.kose@erdogan.edu.tr (T.K.); samet.uygun@erdogan.edu.tr (S.U.); 3 Department of Medical Services and Techniques, School of Health Care Services Vocational, Recep Tayyip Erdoğan University, 53020 Rize, Turkey; kerimali.akyildiz@erdogan.edu.tr; 4 Department of Biochemistry, Faculty of Medicine, Recep Tayyip Erdoğan University, 53020 Rize, Turkey; adnan.yilmaz@erdogan.edu.tr

**Keywords:** periodontal disease, malocclusion, DAI, RANKL, OPG, oxidative stress

## Abstract

**Background:** This study aims to investigate the relationship between malocclusion severity and periodontal clinical parameters and periodontal disease-related biochemical markers in adolescents. **Materials and Methods:** The study’s registration number is NCT07127744. It included 88 individuals aged 12–17 years. Participants were divided into 4 different groups (n = 22) with equal distribution in each group according to the Dental Aesthetic Index (DAI). Clinical (PI, GI, BOP, PPD) and biochemical (RANKL, OPG, RANKL/OPG, TOS, TAS, OSI in gingival crevicular fluid) measurements were performed. Sociodemographic data (age, gender, education, BMI) were recorded. The relationship of all parameters with DAI and with each other was statistically analyzed. Multiple comparison tests, correlation analysis, and multiple linear regression analysis were applied. Statistical significance was accepted at *p* < 0.05. **Results:** Significant differences were found between minor and very severe malocclusion in all clinical periodontal and biochemical parameters (except TAS) (*p* < 0.05). With increasing education level, a decrease in BOP (*p* = 0.041) and a significant increase in OPG (*p* = 0.003) were observed. A strong positive correlation was found between DAI and TOS and RANKL; and a moderate correlation was found with periodontal parameters except PI. Multivariate general linear model analysis revealed that DAI score was significantly associated with all clinical and biochemical parameters (Pillai’s Trace = 0.652, *p* < 0.001). The largest effect sizes were observed for TOS (partial η^2^ = 0.455), RANKL (partial η^2^ = 0.402), and PI (partial η^2^ = 0.300). **Conclusions:** There is a significant association between increased malocclusion severity and periodontal disease-related parameters. TOS and RANKL may be sensitive biomarkers of malocclusion-related periodontal tissue destruction.

## 1. Introduction

Malocclusion is a disorder caused by the teeth and/or jaws being misaligned, crowded, or out of alignment with each other [1]. Globally, it affects approximately 56% of individuals under the age of 18 [2]. The World Dental Federation has stated that malocclusion can increase the prevalence of oral health conditions such as tooth decay, dental trauma, and periodontitis [3]. The Dental Aesthetic Index (DAI) is a simple and reliable index that objectively assesses the aesthetic and functional severity of malocclusion. The index numerically evaluates parameters such as dental crowding, overjet, anterior open bite, and posterior crossbite, and classifies individuals’ orthodontic treatment needs [4].

Periodontal diseases are a group of diseases that primarily include gingivitis, a reversible inflammatory condition characterized by redness, swelling, and bleeding of the gums, and periodontitis, which results in irreversible clinical attachment loss [5]. Gingivitis is the most common periodontal disease, especially in children and adolescents [6]. Gingivitis caused by dental biofilm is quite common among non-adults [7,8]. The relationship between orthodontic conditions and periodontal health is clinically relevant and multifaceted. Malocclusion may influence plaque retention and oral hygiene accessibility, potentially contributing to gingival inflammation and unfavorable periodontal parameters [9,10]. Conversely, orthodontic treatment itself may transiently alter the periodontal environment through tooth movement, changes in microbial composition, and mechanical forces acting on the periodontal tissues.

Although epidemiological studies have emphasized that malocclusion increases the risk of periodontal disease [11,12,13,14,15,16], to our knowledge, there are no studies that objectively establish a relationship between malocclusion severity and the status of periodontal disease based on currently accepted biomarkers of periodontal tissue destruction. Although previous studies have investigated RANKL/OPG or oxidative stress markers in the context of orthodontic tooth movement and treatment-related biological changes [17,18,19,20,21,22,23,24,25,26,27,28,29,30], evidence regarding the association between baseline malocclusion severity and these periodontal and biochemical markers before orthodontic treatment remains limited. Therefore, whether the severity of pre-existing malocclusion is associated with periodontal inflammatory status and related biochemical markers independently of orthodontic treatment remains insufficiently explored.

In this context, this cross-sectional study was planned based on the hypothesis that greater malocclusion severity may be associated with the current bone remodeling markers, Receptor Activator of NF-kappaB Ligand (RANKL) and Osteoprotegerin (OPG) and oxidative stress. This cross-sectional study, in which malocclusion was graded using DAI, is, to our knowledge, the first study to objectively reveal the relationship between malocclusion severity and periodontal tissue destruction through the analysis of the aforementioned biomarkers.

## 2. Materials and Methods

### 2.1. Ethical Approval

This cross-sectional study was conducted between 15 September 2025, and 26 March 2026, with the participation of 88 individuals (47 female, 41 male) aged 12–17 years who attended the Department of Periodontology, Faculty of Dentistry, Recep Tayyip Erdoğan University (RTEU) and met the predefined inclusion and exclusion criteria. The study was previously registered on ClinicalTrials.gov (NCT07127744). It was approved by the RTEU Non-Interventional Ethics Committee (2025/350). The study was conducted in accordance with the principles of the World Medical Association’s Helsinki Declaration. The study details were explained to the patients and their parents/guardians, and their verbal and written consent was obtained.

### 2.2. Sample Size Calculation

The power analysis of the study was performed using G*Power (ver. 3.1.9.7)* software. Based on an effect size of 0.4, a type-1 error level of 0.05, and 85% power, the minimum sample size was determined to be 84 patients, with at least 21 patients in each group. Accordingly, a total of 88 participants were included, 22 from each group.

### 2.3. Study Groups

The study included non-smoker patients aged 12–17 years who had not received periodontal treatment or antioxidant supplementation in the last 6 months, were not using chronic medications affecting periodontal tissues (e.g., cyclosporine A, phenytoin), did not have an active infectious disease, had not taken antibiotics, steroids and/or non-steroidal anti-inflammatory drugs in the last 3 weeks, had all permanent teeth erupted, and required orthodontic treatment. The presence of any congenital craniofacial deformity (genital cleft lip and palate or any craniofacial syndrome or deformity), systemic disease, mental illness, immunosuppressive drugs or immunodeficiency, acute illness, and patients who had previously started or completed orthodontic treatment were excluded.

Patients included in the study were divided into 4 groups according to the DAI scoring system, which determines the severity of orthodontic malocclusion and the need for treatment: DAI-1 (n = 22), DAI-2 (n = 22), DAI-3 (n = 22) and DAI-4 (n = 22).

### 2.4. Dental Aesthetics Index

DAI is an index system that evaluates incisor, canine, and premolar tooth loss, anterior crowding, spacing, diastema, anterior maxillary and mandibular discordance, anterior maxillary and mandibular overjet, vertical anterior open bite, and antero-posterior molar relationship in the dental arch. When calculating the DAI, the score for each component is multiplied by its corresponding magnitude coefficient. The weighted scores are then summed, and the constant value of 13 is added to obtain the final DAI score [31] (Table 1).

To assess the need for orthodontic treatment based on the DAI score, patients are divided into four groups for evaluation [31] (Table 1).

### 2.5. Clinical Periodontal Measurements

Participants’ plaque index (PI) [32], gingival index (GI) [33], bleeding on probing index (BOP) [34] and probing pocket depth (PPD) [35] measurements were performed using a Williams probe (Hu-Friedy, Chicago, IL, USA) by a calibrated examiner using a standardized probing technique. The Williams probe was selected because it was the routinely available periodontal probe used for clinical periodontal examinations at our institution. Measurements were recorded to the nearest millimeter according to the probe markings. Measurements were taken from all four surfaces (mesial, distal, buccal, and lingual/palatal) of each tooth to assess the amount of microbial deposits accumulating on the tooth surface [32], to measure the degree of gingival inflammation [33] and to determine if bleeding occurred during probing [34]. PPD was measured at six different points on each tooth (mesio-buccal, mid-buccal, disto-buccal, mesio-lingual, mid-lingual, and disto-lingual), based on the distance between the deepest point of the pocket and the gingival margin [35]. The obtained values were divided by the total value of all teeth. Results were expressed as scores for PI and GI, in millimeters for PPD, and as a percentage for BOP.

### 2.6. Examiner Calibration

In 20 patients (5 patients per group) not included in the study for clinical periodontal measurements and DAI score determination, training and calibration were performed at different times (baseline and day 7) by the periodontist (N.Y.) and orthodontist (S.U.). Intraclass correlation was found to be 0.982 for PPD and 0.988 for DAI score. This was assessed with a 0.99 confidence interval (IC) and was considered to be excellent reproducibility.

### 2.7. Gingival Crevicular Fluid (GCF) Sampling

To ensure standardization, the canine teeth in each quadrant of each patient, or lateral teeth if canine teeth were unavailable, were isolated using a cotton roll. To prevent contamination, supragingival plaque was gently removed from the relevant teeth, and the teeth were air-dried. GCF samples were collected by inserting PerioPaper strips into the mesiobuccal gingival sulcus to a depth of 1 mm for 30 s. Subsequently, GCF volume was measured using a calibrated instrument (Peritron 8010, Oraflow Inc., New York, NY, USA). PerioPaper strips were placed in 250 μL of phosphate buffer solution and stored in a −80 °C freezer (Thermo Fisher Scientific, Waltham, MA, USA) for biochemical measurements.

### 2.8. Biochemical Analysis

The collected samples were thawed gradually, first at −20 °C and then at +4 °C, after being removed from −80 °C 24 h prior to analysis. On the day of analysis, the samples were brought to room temperature and vortexed. Biochemical analyses were performed at the Medical Biochemistry Research and Application Laboratory of RTEU Faculty of Medicine using enzyme-linked immunosorbent methods (ELISA). Human RANKL (BT LAB, Shanghai, China; Cat. No: e0620Hu) and Human OPG (BT LAB, Shanghai, China; Cat. No: e1558Hu) commercial kits were used. Results were expressed in pg/mL for RANKL and ng/mL for OPG. The RANKL/OPG ratio was also calculated. Total antioxidant status (TAS) and total oxidant status (TOS) levels were measured using Rel Assay (Mega Medical Industry and Trade Ltd., Sarıyer, Turkey) commercial test kits [36,37]. Oxidative stress index (OSI) values were calculated using the formula OSI = (TOS (µmol H_2_O_2_ eq.)/TAS (µmol Trolox eq.)) × 100 [38].

### 2.9. Statistical Analysis

Data analysis was performed using IBM SPSS Statistics version 26. For variables showing normal distribution, the Independent Sample *t*-test was used for comparisons between two groups, and One-way ANOVA was used for comparisons between more than two groups. For variables not showing normal distribution, the Mann–Whitney U test (two groups) and the Kruskal–Wallis H test (more than two groups) were applied. Multiple comparison tests were performed to determine which groups showed significant differences. Tukey HSD was used when variances were homogeneous, and Tamhane T2 test was used when variances were not homogeneous. In cases where a significant difference was found after the Kruskal–Wallis test, pairwise comparisons were made with Benjamini–Hochberg False Discovery Rate (FDR) correction to control for Type I errors. Spearman correlation analysis was used to determine the direction and strength of the relationships between variables. To evaluate the independent contribution of malocclusion severity on clinical and bio-chemical parameters, a multivariate general linear model (GLM) was performed with DAI score as the independent variable and PI, GI, BOP, PPD, RANKL, OPG, TOS, and TAS as dependent variables. For the GLM, Pillai’s Trace was used as the multivariate test statistic, which inherently accounts for multiple dependent variables. To control for Type I errors due to multiple comparisons, the Benjamini–Hochberg FDR correction was applied to post hoc pairwise comparisons and correlation analyses. Statistical significance was accepted at *p* < 0.05 (FDR-adjusted *p*-value). Statistical significance was considered to be *p* < 0.05 for all tests.

## 3. Results

### 3.1. Demographic Data

The chi-square test results for gender, education level, and Body Mass Index (BMI), and the ANOVA results for age, are presented in Table 2 for the DAI groups. No statistically significant differences were found between the DAI groups and the participants’ gender, education level, BMI, and age (Table 2) (Supplementary Table S1).

### 3.2. Comparison of Clinical Periodontal and Biochemical Parameters According to DAI Groups

Significant differences were found among all groups at the PI, RANKL, and TOS levels (*p* < 0.001). There was no significant difference between the DAI-2 and DAI-3 groups in the GI and PPD parameters (*p* > 0.05). While no statistically significant difference was observed between the first three groups in terms of BOP and PPD (*p* > 0.05); the difference was significant between DAI-1 and DAI-4 (*p* < 0.05). Additionally, significant differences were observed in the highest malocclusion severity and in the PPD, OPG, RANKL/OPG, and OSI levels compared to the first two groups (*p* < 0.05). TAS did not show a significant difference among all groups (*p* > 0.05) (Table 3).

### 3.3. Comparison of Clinical Periodontal and Biochemical Parameters According to Gender, Education, and BMI

Age, BMI, and all periodontal and biochemical parameters showed similar results for both genders (*p* > 0.05). While a significant difference was observed between age and education level (*p* < 0.001), high school students had lower bleeding during probing and higher BMI and OPG values compared to middle school students (*p* < 0.05). Statistically significant differences were observed between underweight individuals and the others based on the age of the participants (*p* < 0.001) (Supplementary Tables S2–S4).

### 3.4. Relationship Levels Between Parameters

Correlation analysis showed that greater DAI scores were strongly positively associated with TOS and RANKL, and moderately-to-strongly positively associated with PI, RANKL/OPG, and OSI. DAI was also moderately positively correlated with the periodontal parameters other than PI; while its correlation with OPG was moderately negative after FDR adjustment; however, the negative correlation between DAI and TAS did not remain statistically significant after FDR correction (*p* > 0.05).

Among the periodontal parameters, PI was strongly positively correlated with the other periodontal parameters, and GI showed a very strong positive correlation with BOP. These associations remained significant after FDR correction. All periodontal parameters were moderately positively correlated with RANKL. RANKL/OPG was strongly positively correlated with PI and moderately-to-strongly positively correlated with the other periodontal parameters after FDR adjustment. In addition, PI, GI, and BOP showed moderate positive correlations with TOS and OSI after FDR adjustment; however, the correlation between BOP and OSI did not remain significant after FDR correction.

Regarding the interrelationships among biochemical markers, RANKL/OPG was very strongly negatively correlated with OPG and strongly positively correlated with RANKL. RANKL and RANKL/OPG showed moderate-to-strong positive correlations with TOS, while OSI was moderately positively correlated with these markers after FDR adjustment. OSI was also very strongly negatively correlated with TAS and strongly positively correlated with TOS. (Table 4).

### 3.5. Association of DAI Score with Periodontal and Biochemical Parameters: General Linear Model Analysis

The multivariate general linear model demonstrated a statistically significant overall association between DAI score and the set of periodontal and biochemical parameters examined (Pillai’s Trace = 0.652, F(8.79) = 18.492, *p* < 0.001), indicating that DAI score was significantly associated with the combined dependent variables. In the subsequent univariate analyses, DAI score was significantly associated with PI (F = 36.913, *p* < 0.001, partial η^2^ = 0.300), GI (F = 20.607, *p* < 0.001, partial η^2^ = 0.193), BOP (F = 24.810, *p* < 0.001, partial η^2^ = 0.224), PPD (F = 20.265, *p* < 0.001, partial η^2^ = 0.191), RANKL (F = 57.865, *p* < 0.001, partial η^2^ = 0.402), OPG (F = 8.266, *p* = 0.005, partial η^2^ = 0.088), TOS (F = 71.810, *p* < 0.001, partial η^2^ = 0.455), and TAS (F = 5.299, *p* = 0.024, partial η^2^ = 0.058). The corresponding regression coefficients indicated positive associations of DAI score with PI, GI, BOP, PPD, RANKL, and TOS, whereas negative associations were observed for OPG and TAS. The proportion of variance explained by DAI score ranged from 5.8% for TAS to 45.5% for TOS. The largest effect sizes were observed for TOS (partial η^2^ = 0.455), RANKL (partial η^2^ = 0.402), and PI (partial η^2^ = 0.300), whereas the association with TAS was comparatively weak (partial η^2^ = 0.058). Overall, higher DAI scores were associated with poorer periodontal parameters and an unfavorable profile of oxidative stress-related biomarkers (Table 5).

## 4. Discussion

Recent studies have shown that malocclusion treatment causes changes in clinical periodontal parameters [10,14,15] as well as GCF RANKL/OPG [19,21] and oxidative stress [24,28]. This study is the first to examine the relationship between malocclusion severity and clinical and biochemical periodontal markers. When the findings are considered together, it is understood that with increasing malocclusion severity, there is a particularly high increase in TOS, while periodontal parameters, RANKL, and the RANKL/OPG ratio show a moderate increase. The negative correlation between OPG and TAS levels and the DAI score suggests that protective mechanisms decrease with increasing malocclusion severity. The lack of correlation between BMI and the examined parameters indicates that BMI is not an independent predictor in this study population. In the multivariate regression analysis, DAI score emerged as a significant independent predictor of all clinical and biochemical parameters, with the strongest effects observed on TOS (partial η^2^ = 0.455) and RANKL (partial η^2^ = 0.402). These findings suggest that malocclusion severity is not only associated with increased plaque accumulation and gingival inflammation but also with elevated oxidative stress and bone resorption markers. The effect sizes for TOS and RANKL were notably larger than those for clinical periodontal parameters, indicating that biochemical markers may be more sensitive to the effects of malocclusion severity.

Although different index systems have been developed to determine the severity of malocclusion, DAI classifies not only the severity of malocclusion but also the need for treatment [31]. Due to its ease of application in clinical practice and its objectivity and reliability, it has found use in recent studies [10,39]. In a study comparing the severity of malocclusion determined by DAI in 12 and 15-year-old children [10], malocclusion and periodontal disease were observed to be more common in 12-year-old individuals. In a study by Tariq et al. [39] which included adolescents, a strong association was found between the presence of malocclusion and periodontal disease (*p* = 0.003). In this study, a significant decrease in BOP scores and a parallel significant increase in OPG, a bone formation marker, were observed in high school students compared to middle school students. Accordingly, the association between malocclusion severity and periodontal status may be particularly relevant in younger age groups. However, there are also studies in the literature reporting that the relationship between DAI score and the need for periodontal treatment is not significant [40] and that malocclusion worsens with increasing age [41].

In the presented study, significant differences were obtained in all clinical periodontal index outcomes, particularly between individuals with the lowest and highest DAI scores. Although PPD differed significantly between DAI groups, the approximately 0.4 mm difference likely reflects the narrow range of probing depths expected in this young population and should therefore be interpreted as a modest association rather than a clinically substantial difference. Our findings demonstrate that severe malocclusion can increase plaque accumulation and, consequently, the degree of gingival inflammation. In a study comparing the severity of malocclusion in two different age groups [10], it was reported that the severity of malocclusion, as measured by the DAI score, was influenced by dental plaque accumulation. Another study in adolescents [15], supports our findings, suggesting that malocclusion severity is strongly associated with gingival bleeding and PPD. In a study of 6–12-year-old children [14], it was stated that gingivitis was associated with overjet (*p* = 0.003) and anterior openbite (*p* = 0.008), but no significant change was observed with other DAI variables. According to a study by Medina-Vega et al. [13] a significant result was obtained between DAI and BOP (*p* = 0.003), but no similar relationship was found with the presence of dental calculus. This study shows parallelism with our study findings in terms of BOP score. However, since PI and the presence of dental calculus are not exactly the same, we believe that evaluating the relationship using PI as examined in our study would be more realistic.

GCF is a fluid that provides quantitative findings of important biomarkers in the diagnosis and monitoring of periodontal disease [42,43]. GCF collection is a frequently used method due to its ease of application and non-invasive nature [42]. Although various biomarkers have been examined using this method, RANKL and OPG, which are involved in bone metabolism, are among them. The bone resorption marker RANKL, its antagonist OPG, and changes in the RANKL/OPG ratio are valuable indicators in the osteoimmunological mechanism of periodontal disease pathogenesis [44,45]. Furthermore, they have been the subject of numerous studies due to their role in the build-and-breakdown cycle that occurs during orthodontic tooth movement [17,18,19,20]. The observed biomarker differences should not be interpreted as evidence of established periodontal tissue destruction, particularly given the young age of the participants and the absence of clinical attachment loss; rather, they may reflect differences in biological processes relevant to periodontal inflammation and bone remodeling. In this study, it is noteworthy that there are significant changes in RANKL levels at every stage where the severity of malocclusion increases. Furthermore, the results of the regression analysis indicate that RANKL is the second most influential marker affecting DAI. The fact that OPG and RANKL/OPG results indicate significant differences particularly between DAI-1 and DAI-4 reflects the gradual transition observed in the other groups. Our findings suggest a possible relationship between the dramatic increase in malocclusion severity and periodontal status in terms of bone remodeling. Studies have focused on changes in RANKL and OPG during orthodontic treatment [21,22,23]. In a model with intermittent vibration force applied with aligners [22], it was reported that no significant change was observed in RANKL and OPG levels in GCF. In contrast, another study [23] reported significant changes in GCF RANKL and OPG levels at different times after surgically assisted rapid maxillary expansion. In particular, the significant increase in the RANKL/OPG ratio during the activation period clearly reveals the changes in bone remodeling during the orthodontic treatment process. However, as a result of a comprehensive literature search, we unfortunately could not find any studies that included bone biomarkers before orthodontic treatment and/or at the diagnostic stage.

Oxidative stress, like bone remodeling, plays an important role in the pathogenesis of periodontal disease [46]. Disruption of the antioxidant-oxidant balance affects periodontal tissues [47]. Similarly, changes in oxidative stress markers during orthodontic tooth movement have been investigated in numerous studies [24,26,27,29,30]. In an observational study [25], pro-oxidant markers were found to be significantly higher in individuals with class 2 malocclusion with varying degrees of overjet compared to healthy controls. The results of the study by Angeles-Estrada et al. [27] highlighted a significant increase in the gene expression of antioxidant enzymes at the sixth month of fixed orthodontic treatment. In a recently published study [30], it was observed that there were significant differences in the levels of 8-OHdG, an oxidative DNA damage marker, in GCF when comparing fixed appliance with clear aligners at the sixth month of treatment. In a study comparing traditional brackets and clear aligners [29] a significant relationship was found between BOP and TOS (*p* = 0.033), while no significant relationship was found with TAS (*p* = 0.603). In this respect, our study findings are consistent. Furthermore, in the presented study, TOS was the most associated marker with malocclusion severity. TAS decreased with malocclusion severity but did not show significant changes. Although the association between malocclusion severity, plaque accumulation, and gingival inflammation is clinically expected, our findings suggest that this relationship may involve biological mechanisms beyond plaque accumulation alone. Notably, in linear model analysis, TOS and RANKL contributed more than PI. This finding highlights the potential relevance of oxidative stress and RANKL as biological markers associated with malocclusion severity and suggests that biochemical alterations may provide information that is not fully captured by conventional clinical plaque measures. These findings should, however, be interpreted as associations rather than causal relationships.

This study investigated the relationship between the severity of malocclusion and periodontal status by evaluating clinical, osteoimmunological, and oxidative factors. To control for Type I errors due to multiple comparisons, the Benjamini–Hochberg FDR correction was applied to correlation analyses. While most associations remained significant after correction, three weak correlations (DAI-TAS, GI-TAS, and BOP-OSI) did not survive the adjustment and should therefore be interpreted with caution as potential trends requiring confirmation in future studies. Importantly, the RANKL/OPG and oxidative stress studies cited above primarily evaluated biological changes associated with orthodontic tooth movement or treatment. Therefore, their findings cannot be directly extrapolated to the relationship between malocclusion severity and periodontal or biochemical status before orthodontic intervention. By evaluating these parameters at baseline, the present study provides a different perspective by examining whether the severity of an existing malocclusion is associated with periodontal and biochemical alterations in the absence of orthodontic treatment. However, this study has some limitations. Firstly, the cross-sectional design precludes causal inference; therefore, the observed associations between malocclusion severity, periodontal parameters, and biochemical markers should not be interpreted as causal relationships. Secondly, information regarding diet, sleep patterns, exam stress, hormonal changes, and brushing frequency and duration—factors that could affect participants’ periodontal status—was not included. The cross-sectional design, together with the absence of detailed oral hygiene behavior data, precluded assessment of whether plaque accumulation mediates or confounds the association between malocclusion severity and periodontal disease-related parameters. Thirdly, while the DAI classification is a frequently preferred system in clinical practice, it is inadequate for diagnosing deep and crossbites. Fourthly, the recruitment of adolescents from a university dental hospital setting may limit the generalizability of the findings to the broader adolescent population. Another limitation is that PPD measurements obtained with a Williams probe rather than a UNC-15 probe may have introduced a small degree of measurement imprecision, although the use of the same probe and standardized probing technique throughout the study ensured measurement consistency. Finally, although grouping is done based on the total DAI score, analyzing its sub-scores along with other parameters would add value to future studies.

## 5. Conclusions

There is a significant correlation between increased malocclusion severity and periodontalstatus. The stronger contribution of TOS and RANKL compared to PI suggests that biochemical markers may provide additional information about biological processes associated with malocclusion severity. Severe malocclusion, in particular, may regulate the periodontal bone resorption process by facilitating plaque accumulation, thereby triggering oxidative stress and RANKL-related osteoclastogenesis.

## Figures and Tables

**Table 1:** Calculating the DAI Score and the Need for Orthodontic Treatment.

No	DAI Component	Description/Scoring	Coefficient
1	Missing teeth (incisors, canines, premolars in dental arch)	Number of missing teeth	6
2	Crowding in incisal segments	0 = none, 1 = one segment, 2 = two segments	1
3	Spacing in incisal segments	0 = none, 1 = one segment, 2 = two segments	1
4	Midline diastema (mm)	Measured in mm	3
5	Maxillary anterior irregularity (mm)	Measured in mm	1
6	Mandibular anterior irregularity (mm)	Measured in mm	1
7	Maxillary overjet (mm)	Measured in mm	2
8	Mandibular overjet (mm)	Measured in mm	4
9	Vertical anterior open bite (mm)	Measured in mm	4
10	Anteroposterior molar relationship	0 = normal, 1 = ½ cusp deviation (mesial/distal), 2 = ≥1 cusp deviation	3
	Constant	Fixed value	13
	Total DAI Score	Sum of (component × coefficient) + constant	
DAI Score		Interpretation	Orthodontic Treatment Need
≤25		Normal or minor malocclusion	No or slight treatment need
26–30		Definite malocclusion	Elective treatment
31–35		Severe malocclusion	Treatment highly desirable
≥36		Very severe (handicapping) malocclusion	Treatment mandatory

**Table 2:** Distribution of gender, education, BMI, and age according to DAI groups.

Variable	Category	DAI-1 n (%)	DAI-2 n (%)	DAI-3 n (%)	DAI-4 n (%)	χ^2^/F	*p*
Gender	Male	12 (29.3)	10 (24.4)	8 (19.5)	11 (26.8)	1.598	0.660
	Female	10 (21.3)	12 (25.5)	14 (29.8)	11 (23.4)		
Education	Middle school	6 (17.6)	10 (29.4)	7 (20.6)	11 (32.4)	3.259	0.353
	High school	16 (29.6)	12 (22.2)	15 (27.8)	11 (20.4)		
BMI (kg/m^2^)	Underweight (<18.5)	3 (14.3)	8 (38.1)	2 (9.5)	8 (38.1)	9.014	0.173
	Normal (18.5–24.9)	15 (29.4)	10 (19.6)	14 (27.5)	12 (23.5)		
	Overweight/Obese (>25)	4 (25.0)	4 (25.0)	6 (37.5)	2 (12.5)		
Age	Mean ±S.D.	14.6 ± 1.7	14.1 ± 1.7	14.73 ± 1.69	14.25 ± 1.65	2.24	0.089

(DAI: Dental Aesthetic Index, BMI: Body Mass Index, n: frequency, S.D.: Standard Deviation, %: percentage, kg: kilogram, m^2^: square meter, X^2^: Chi-square analysis, F: One-way ANOVA, *p* < 0.05 statistically significant).

**Table 3:** Comparison of Clinical Periodontal and Biochemical Parameters According to DAI Groups.

	DAI-1 (n = 22)	DAI-2 (n = 22)	DAI-3 (n = 22)	DAI-4 (n = 22)	
Parameter	Mean ± S.D. (Med.)	Mean ± S.D. (Med.)	Mean ± S.D. (Med.)	Mean ± S.D. (Med.)	*p*
PI	1.04 ± 0.50 (0.99) ^a^	1.36 ± 0.44 (1.49) ^b^	1.58 ± 0.50 (1.53) ^c^	1.83 ± 0.49 (1.94) ^d^	<0.001 **
GI	0.44 ± 0.38 (0.41) ^a^	0.76 ± 0.52 (0.73) ^b^	0.79 ± 0.46 (0.72) ^b^	1.01 ± 0.61 (0.88) ^c^	0.004 *
BOP (%)	15.45 ± 12.68 (15) ^a^	21.36 ± 14.93 (21) ^ab^	23.27 ± 12.51 (21) ^ab^	31.77 ± 18.72 (29) ^b^	0.006 *
PPD (mm)	1.66 ± 0.34 (1.64) ^a^	1.69 ± 0.32 (1.71) ^a^	1.80 ± 0.48 (1.69) ^ab^	2.03 ± 0.41 (2.02) ^b^	0.010 *
RANKL (pg/mL)	208.6 ± 36.1 (195.3) ^a^	237.2 ± 39.3 (249.8) ^b^	269.2 ± 52.35 (275.7) ^c^	309.7 ± 54.6 (283.6) ^d^	<0.001 **
OPG (ng/mL)	7.66 ± 2.69 (7.62) ^c^	6.82 ± 2.15 (7.05) ^bc^	5.93 ± 2.28 (5.71) ^ab^	5.55 ± 2.05 (5.23) ^a^	0.014 *
RANKL/OPG	33.21 ± 21.83 (28.3) ^a^	40.93 ± 25.49 (33.2) ^ab^	62.82 ± 63.37 (47.9) ^bc^	64.37 ± 30.87 (55.9) ^c^	<0.001 **
TOS (U/mL)	16.60 ± 3.13 (16.94) ^a^	20.32 ± 3.66 (18.82) ^b^	22.87 ± 3.96 (21.91) ^c^	26.58 ± 4.08 (27.37) ^d^	<0.001 **
TAS (U/mL)	3.04 ± 1.30 (2.76)	2.78 ± 1.10 (2.40)	2.54 ± 1.15 (2.22)	2.27 ± 0.82 (2.16)	0.127
OSI	6.71 ± 3.89 (5.48) ^a^	8.27 ± 2.99 (8.12) ^ab^	12.69 ± 11.64 (10.14) ^bc^	13.72 ± 7.02 (12.33) ^c^	<0.001 **

(DAI: Dental Aesthetic Index, PI: Plaque Index, GI: Gingival Index, BOP: Bleeding on Probing, PPD: Probing Pocket Depth, RANKL: Receptor Activator of NF-kappaB Ligand, OPG: Osteoprotegerin, TOS: Total Oxidant Status, TAS: Total Antioxidant Status, OSI: Oxidative Stress Index, %: Percentage, mm: millimeter, pg: picogram, ng: nanogram, mL: milliliter, U: Unit, S.D.: Standard Deviation, Med.: Median, *: *p* < 0.05, **: *p* < 0.001. Different letter combinations (a, b, c, d) indicate statistically significant differences between groups (*p* < 0.05). There is no significant difference between groups with the same letter. One-way ANOVA and Tukey HSD post hoc tests were used for normally distributed variables. The Tamhane T2 post hoc test was applied for the GI variable, where homogeneity of variance was not achieved. The Kruskal–Wallis test was used for non-normally distributed variables (RANKL/OPG, OSI). Post hoc pairwise comparisons were performed with Benjamini–Hochberg False Discovery Rate (FDR) correction to control for Type I errors across multiple comparisons (*p* < 0.05). All significant differences remained significant after FDR correction due to strong effect sizes).

**Table 4:** Relationship Levels Between Parameters.

Variable	1	2	3	4	5	6	7	8	9	10	11
1. DAI	—										
2. PI	0.52 **	—									
3. GI	0.37 **	0.78 **	—								
4. BOP (%)	0.34 **	0.68 **	0.80 **	—							
5. PPD (mm)	0.33 **	0.70 **	0.56 **	0.46 **	—						
6. RANKL (pg/mL)	0.64 **	0.37 **	0.23 *	0.23 *	0.28 **	—					
7. OPG (ng/mL)	−0.39 **	−0.62 **	−0.50 **	−0.39 **	−0.36 **	−0.29 **	—				
8. RANKL/OPG	0.56 **	0.63 **	0.48 **	0.40 **	0.42 **	0.69 **	−0.86 **	—			
9. TOS (U/mL)	0.70 **	0.37 **	0.31 **	0.30 **	0.17	0.42 **	−0.34 **	0.42 **	—		
10. TAS (U/mL)	−0.27	−0.13	−0.22	−0.07	−0.06	−0.06	0.06	−0.06	−0.20	—	
11. OSI	0.54 **	0.28 **	0.33 **	0.23	0.14	0.26 *	−0.18	0.24 *	0.64 **	−0.84 **	—
12. BMI (kg/m^2^)	−0.06	0.01	−0.00	0.01	−0.06	0.01	0.07	−0.06	−0.04	−0.03	−0.01

(DAI: Dental Aesthetic Index, PI: Plaque Index, GI: Gingival Index, BOP: Bleeding on Probing, PPD: Probing Pocket Depth, RANKL: Receptor Activator of NF-kappaB Ligand, OPG: Osteoprotegerin, TOS: Total Oxidant Status, TAS: Total Antioxidant Status, OSI: Oxidative Stress Index, BMI: Body Mass Index, %: Percentage, mm: millimeter, pg: picogram, ng: nanogram, ml: milliliter, U: Unit, kg: kilogram, m^2^: square meters. Blue tones have a positive correlation, while orange-red tones have a negative correlation; color intensity reflects the magnitude of the correlation. Positive (blue): weak (0.00–0.20) → moderate (0.20–0.40) → moderate-strong (0.40–0.60) → strong (0.60–0.80) → very strong (0.80–1.00), Negative (orange/red): same ranges in the opposite direction. Spearman correlation analysis was performed. Statistical significance was determined after Benjamini–Hochberg False Discovery Rate correction for multiple comparisons. *: *p* < 0.05, **: *p* < 0.001).

**Table 5:** Association of DAI Score with Periodontal and Biochemical Parameters: General Linear Model Analysis.

Dependent Variable	B	SE	t	p	95% CI	R^2^	Adjusted R^2^
PI	0.034	0.006	6.076	<0.001	0.023–0.045	0.300	0.292
GI	0.026	0.006	4.540	<0.001	0.015–0.038	0.193	0.184
BOP	0.839	0.168	4.981	<0.001	0.504–1.173	0.224	0.215
PPD	0.020	0.004	4.502	<0.001	0.011–0.029	0.191	0.181
RANKL	4.209	0.553	7.607	<0.001	3.109–5.309	0.402	0.395
OPG	−0.080	0.028	−2.875	0.005	−0.135–−0.025	0.088	0.077
TOS	0.392	0.046	8.474	<0.001	0.300–0.484	0.455	0.449
TAS	−0.030	0.013	−2.302	0.024	−0.057–−0.004	0.058	0.047

(DAI score was entered as the independent variable in the general linear model. B, unstandardized regression coefficient; SE, standard error; CI, confidence interval; R^2^, coefficient of determination. PI: Plaque Index; GI: Gingival Index; BOP: Bleeding on Probing; PPD: Probing Pocket Depth; RANKL: Receptor Activator of Nuclear Factor kappa-B Ligand; OPG: Osteoprotegerin; TOS: Total Oxidant Status; TAS: Total Antioxidant Status. Statistical significance was set at *p* < 0.05).

## Data Availability

The data that support the findings of this study are available from the corresponding author upon reasonable request.

## Supplementary Materials

The following supporting information can be downloaded at: https://www.mdpi.com/article/10.3390/diagnostics16182929/s1. Table S1: Descriptive statistics of clinical periodontal parameters and biochemical markers; Table S2: Comparison of Demographic, Clinical Periodontal, and Biochemical Parameters by Gender; Table S3: Comparison of Demographic, Clinical Periodontal, and Biochemical Parameters by Education; Table S4: Comparison of Demographic, Clinical Periodontal, and Biochemical Parameters by BMI.

## Abbreviations

BMI	Body Mass Index
BOP	Bleeding on Probing Index
DAI	Dental Aesthetic Index
GCF	Gingival Crevicular Fluid
GI	Gingival Index
OPG	Osteoprotegerin
OSI	Oxidative Stress Index
PI	Plaque Index
PPD	Probing Pocket Depth
RANKL	Receptor Activator of NF-kappaB Ligand
TAS	Total Antioxidant Status
TOS	Total Oxidant Status
