# Supplementary material for: The Relationship Between Orthodontic Malocclusion Severity and Periodontal Clinical and Biochemical Parameters in Adolescents: A Cross-Sectional Study
Source: Diagnostics (Basel). 2026 Sep 10;16(18):2929. doi: 10.3390/diagnostics16182929 (PMC13605200; doi:10.3390/diagnostics16182929)
Supplement: Supplementary file 1 [file diagnostics-16-02929-s001.zip › diagnostics-4473384-Supplementary Tables.pdf]

Supplementary Table S1. Descriptive statistics of clinical periodontal parameters and biochemical markers

Variable	Mean±S.D.	Median	Minimum	Maximum
**DAI**	32.20 ± 8.90	30.50	16.50	69.00
**PI**	1.45 ± 0.56	1.46	0.23	2.77
**GI**	0.75 ± 0.53	0.66	0.00	2.02
**BOP (%)**	23.00 ± 16.00	21.00	0.00	72.00
**PPD (mm)**	1.79 ± 0.41	1.74	1.05	2.80
**RANKL (pg/ml)**	256.17 ± 59.14	260.74	157.36	395.69
**OPG (ng/ml)**	6.49 ± 2.41	6.33	1.20	12.77
**RANKL/OPG**	50.33 ± 40.70	39.58	14.87	256.94
**TOS (U/ml)**	21.59 ± 5.18	21.72	10.15	35.35
**TAS (U/ml)**	2.66 ± 1.12	2.31	0.49	5.73
**OSI**	10.35 ± 7.69	9.23	2.71	50.28

(DAI: Dental Aesthetic Index, PI: Plaque Index, GI: Gingival Index, BOP: Bleeding on Probing, PPD: Probing Pocket Depth, RANKL: Receptor Activator of NF-kappaB Ligand, OPG: Osteoprotegerin, TOS: Total Oxidant Status, TAS: Total Antioxidant Status, OSI: Oxidative Stress Index, %: Percentage, mm: millimeter, pg: picogram, ng: nanogram, ml: milliliter, U: Unit, S.D.: Standard Deviation)

Supplementary Table S2. Comparison of Demographic, Clinical Periodontal, and Biochemical Parameters by Gender

Parameter	Male (n=41)	Female (n=47)	p
	Mean±S.D.(Med.)	Mean±S.D.(Med.)	
**Age (year)**	14.17±1.66(14.00)	14.32±1.67(14.00)	0.677
**BMI (kg/m^2)**	22.16±4.23(21.26)	20.62±3.40(19.53)	0.062
**DAI**	31.60±8.72(31.60)	32.75±9.13(32.75)	0.550
**PI**	1.45±0.55(1.48)	1.45±0.57(1.43)	0.979
**GI**	0.77±0.54(0.67)	0.73±0.52(0.66)	0.697
**BOP (%)**	21.32±15.69(17)	24.40±15.92(22)	0.363
**PPD (mm)**	1.80±0.43(1.75)	1.79±0.40(1.73)	0.940
**RANKL (pg/mL)**	263.87±60.35(267.98)	249.46±57.87(251.99)	0.257
**OPG (ng/mL)**	6.53±2.65(6.21)	6.46±2.21(6.62)	0.899
**RANKL/OPG**	51.58±40.19(41.48)	49.24±41.55(37.94)	0.313$^{\#}$
**TOS (U/mL)**	20.79±4.77(19.55)	22.29±5.47(22.55)	0.176
**TAS (U/mL)**	2.51±0.99(2.19)	2.79±1.22(2.40)	0.253
**OSI**	9.66±4.75(8.51)	10.95±9.56(9.52)	0.812$^{\#}$

(BMI: Body Mass Index, DAI: Dental Aesthetic Index, PI: Plaque Index, GI: Gingival Index, BOP: Bleeding on Probing, PPD: Probing Pocket Depth, RANKL: Receptor Activator of NF-kappaB Ligand, OPG: Osteoprotegerin, TOS: Total Oxidant Status, TAS: Total Antioxidant Status, OSI: Oxidative Stress Index, %: Percentage, mm: millimeter, pg: picogram, ng: nanogram, ml: milliliter, U: Unit, S.D.: Standard Deviation, Med.: Median, n: frequency, *$p<0.05$. The independent t-test was used for normally distributed variables, and the Mann-Whitney U test was used for non-normally distributed variables ($^{\#}$).)

Supplementary Table S3. Comparison of Demographic, Clinical Periodontal, and Biochemical Parameters by Education

Parameter	Middle school (n=34)	High School (n=54)	p
	Mean±S.D.(Med.)	Mean±S.D.(Med.)	
**Age (year)**	12.53±0.71(12.00)	15.33±1.05(15.00)	**<0.001****
**BMI (kg/m²)**	20.32±4.26(19.25)	21.97±3.49(20.83)	**0.049***
**DAI**	33.88±10.20(33.88)	31.16±7.91(31.16)	0.164
**PI**	1.53±0.55(1.53)	1.40±0.56(1.40)	0.320
**GI**	0.85±0.56(0.69)	0.68±0.50(0.66)	0.155
**BOP (%)**	27.29±16.93(26.50)	20.24±14.54(19.00)	**0.041***
**PPD (mm)**	1.82±0.44(1.72)	1.78±0.39(1.76)	0.669
**RANKL (pg/mL)**	252.35±59.82(251.97)	258.58±59.14(265.96)	0.633
**OPG (ng/mL)**	5.55±1.88(5.71)	7.08±2.53(6.62)	**0.003***
**RANKL/OPG**	57.89±45.31(47.28)	45.57±37.17(37.49)	0.080#
**TOS (U/mL)**	21.74±5.43(21.90)	21.50±5.06(21.42)	0.836
**TAS (U/mL)**	2.72±1.15(2.31)	2.62±1.11(2.31)	0.666
**OSI**	9.47±4.96(8.84)	10.90±9.00(9.34)	0.797#

(BMI: Body Mass Index, DAI: Dental Aesthetic Index, PI: Plaque Index, GI: Gingival Index, BOP: Bleeding on Probing, PPD: Probing Pocket Depth, RANKL: Receptor Activator of NF-kappaB Ligand, OPG: Osteoprotegerin, TOS: Total Oxidant Status, TAS: Total Antioxidant Status, OSI: Oxidative Stress Index, kg: kilogram, m^2: square meters, %: Percentage, mm: millimeter, pg: picogram, ng: nanogram, ml: milliliter, U: Unit, S.D.: Standard Deviation, Med.: Median, n: frequency, *p<0.05, **p<0.001. The independent t-test was used for normally distributed variables, and the Mann-Whitney U test was used for non-normally distributed variables (#).)

Supplementary Table S4. Comparison of Demographic, Clinical Periodontal, and Biochemical Parameters by BMI

Parameter	Underweight (<18.5) (n=21)	Normal (18.5-24.9) (n=51)	Overweight/Obese (>25) (n=16)	p
	**Mean±S.D.(Med.)**	**Mean±S.D.(Med.)**	**Mean±S.D.(Med.)**	
**Age (year)**	12.90±1.14(12.90)[a]	14.63±1.52(14.63)[b]	14.81±1.76(14.81)[b]	**<0.001****
**PI**	1.55±0.51(1.62)	1.41±0.57(1.38)	1.45±0.58(1.53)	0.649
**GI**	0.79±0.54(0.64)	0.73±0.52(0.67)	0.74±0.57(0.54)	0.897
**BOP (%)**	24.71±15.58(23)	22.59±16.01(20)	21.88±16.22(20)	0.837
**PPD (mm)**	1.83±0.44(1.72)	1.81±0.40(1.78)	1.70±0.43(1.50)	0.576
**RANKL (pg/mL)**	264.1±61.4(266.4)	253.36±55.8(259.98)	254.69±68.90(258.7)	0.780
**OPG (ng/mL)**	5.93±1.89(6.02)	6.60±2.50(6.62)	6.90±2.72(6.54)	0.432
**RANKL/OPG**	52.10±28.72(46.78)	52.04±49.08(38.35)	42.58±20.05(32.90)	0.522[#]
**TOS (U/mL)**	22.41±5.33(22.73)	21.03±5.32(21.18)	22.30±4.58(21.71)	0.499
**TAS (U/mL)**	2.69±1.21(2.31)	2.66±1.17(2.32)	2.60±0.90(2.19)	0.967
**OSI**	10.24±6.78(9.32)	10.67±9.00(8.16)	9.47±3.27(10.36)	0.771[#]

(DAI: Dental Aesthetic Index, GI: Gingival Index, BOP: Bleeding on Probing, PPD: Probing Pocket Depth, RANKL: Receptor Activator of NF-kappaB Ligand, OPG: Osteoprotegerin, TOS: Total Oxidant Status, TAS: Total Antioxidant Status, OSI: Oxidative Stress Index, %: Percentage, mm: millimeter, pg: picogram, ng: nanogram, ml: milliliter, U: Unit, S.D.: Standard Deviation, Med.: Median, n: frequency, *p<0.05, **p<0.001. One-way ANOVA was used for normally distributed variables, and Kruskal-Wallis test was used for non-normally distributed variables ([#]). Since the variances in the age variable were homogeneous, the Tukey multiple comparison test was used. Different letter groups (a, b) show statistically significant differences (p<0.05). There is no significant difference between groups carrying the same letter.)
